# Antioxidant Defense Strategies Against *Diaporthe eres* Infection in Hongyang Kiwifruit

**DOI:** 10.3390/biology14091169

**Published:** 2025-09-02

**Authors:** Lizhen Ling, Tao Yang, Xiaoqing Long, Shengyu Pan, Shudong Zhang

**Affiliations:** Key Laboratory City for Study and Utilization of Ethnic Medicinal Plant Resources of Western Guizhou Province, Liupanshui Normal University, Liupanshui 553004, China; primula_ling@foxmail.com (L.L.); pnshengyu@foxmail.com (S.P.)

**Keywords:** kiwifruit, soft rot disease, *Diaporthe*, antioxidant enzyme

## Abstract

*Diaporthe eres* is a harmful pathogen affecting Hongyang kiwifruit (*Actinidia chinensis*) after harvest, yet the antioxidant defense strategies are not well understood. This research thoroughly examines the dynamics of the antioxidant response during the infection process. Significant findings indicate an initial 3-day latent period (0–3 dpi) that allowed for pathogen establishment, followed by irreversible tissue breakdown characterized by water-soaked lesions at 4 dpi. The study identified a biphasic activation pattern of superoxide dismutase (SOD) with dual activity peaks (1 dpi and 4 dpi), orchestrated by mitochondrial hub gene *CEY00_Acc02790* that coordinates peroxidase (POD) networks, while peroxidase (POD) activity exhibited a synchronized but temporary increase, peaking at 4 dpi. Further bioinformatic analysis revealed the possible functional specialization of POD isoforms: α-helix-rich extracellular variants drove cell wall reinforcement through lignification, while random coil-dominant intracellular variants formed to mitigate cytoplasmic reactive oxygen species (ROS) damage, establishing dual physicochemical barriers. Malondialdehyde (MDA) levels rose significantly by 3 dpi, indicating permanent membrane damage. Collectively, these findings elucidate the mechanistic foundation of the *Actinidia*–*Diaporthe* pathosystem, identifying the bimodal SOD response and POD specialization as prime targets for developing resistant cultivars and precision postharvest interventions, ultimately reducing losses through biochemical interception of pathogenesis.

## Simple Summary

This study investigated how Hongyang kiwifruit defends against postharvest infection by the harmful fungus *Diaporthe eres* through its antioxidant responses. Our findings show the fungus establishes itself during a hidden 3-day period before causing visible decay symptoms (water-soaked lesions) on day 4. The fruit fights back primarily using the antioxidant enzyme superoxide dismutase (SOD), which showed two distinct waves of increased activity and gene expression levels (peaking at days 1 and 4). Peroxidase (POD) activity also rose temporarily but less effectively. A significant marker of cell membrane damage (malondialdehyde, MDA) increased dramatically by day 3, indicating permanent harm. This suggests that the fruit’s initial, coordinated antioxidant defense is crucial but eventually overwhelmed. The loss of membrane integrity triggers the visible rotting. Understanding this process, especially the unique two-peak SOD response, offers new insights for developing kiwifruit varieties resistant to this pathogen and better methods to reduce storage losses.

## 1. Introduction

Kiwifruit (*Actinidia* spp.) represents a horticultural crop of significant economic importance [1]. China has abundant genetic resources, in which 52 out of 54 species are found [2,3]. Kiwifruit is valued for its distinctive taste, texture, rich vitamin C, dietary fiber, potassium, folate, antioxidants, and various other nutrients that enhance immune function, support digestive health, and help prevent heart disease [2,4,5,6]. The Hongyang kiwifruit, a unique red-fleshed variety from China, has become a key player in industrial advancement due to its exceptional nutritional profile and market potential [1]. However, this variety is particularly vulnerable to soft rot during storage after harvest [7]. Studies have identified *Botryosphaeria dothidea* and *Diaporthe* spp. as the main culprits [8,9,10,11], leading to annual economic losses of 30–40% [3]. In recent years, researchers have explored eco-friendly preservation methods to combat kiwifruit soft rot. For instance, Zhang et al. investigated the effects of extracts from traditional Chinese herbs like star anise, prickly ash, *Roxburgh rose*, and *Incarvillea arguta* on *Phomopsis* spp., discovering that these natural compounds can significantly inhibit pathogen growth at specific concentrations [12]. However, the disease still occurs even at the maximum inhibitory levels, although lesions may be smaller [13]. Therefore, it is necessary to understand the Hongyang kiwifruit-pathogen interactions for developing effective control strategies.

Upon pathogen recognition, plants trigger an immediate oxidative burst characterized by rapid accumulation of reactive oxygen species (ROS), which serves as a critical signaling hub for activating defense responses, including the hypersensitive reaction (HR) —a localized programmed cell death that restricts biotrophic pathogen spread [14,15]. This biochemical cascade initiates with plasma membrane-bound NADPH oxidases (RBOHs) generating primary superoxide radicals (O_2_^−^), which are subsequently converted to hydrogen peroxide (H_2_O_2_) through the catalytic action of superoxide dismutases (SODs) [16,17]. These metalloenzymes exhibit compartment-specific localizations and functions: MnSODs neutralize mitochondrial/peroxisomal ROS derived from respiratory processes, FeSODs and chloroplastic Cu/ZnSODs scavenge photosynthetic byproducts in plastids, while cytosolic/nuclear Cu/ZnSODs regulate redox signaling networks. For necrotrophic pathogens like *Botrytis cinerea*, however, this oxidative response is strategically exploited to induce host cell death and access nutrients, transforming a defensive mechanism into a pathogenic vulnerability [15].

The H_2_O_2_ generated by SOD activity is metabolized by class III peroxidases (POD)—secretory glycoproteins localized predominantly in the apoplast and vacuoles [18]. These enzymes perform dual functions: they not only eliminate H_2_O_2_ through oxidation of phenolic/indolic compounds but also execute defensive lignification and suberin deposition via cell wall reinforcement [18,19]. When sustained ROS production overwhelms these antioxidant systems, uncontrolled H_2_O_2_ accumulation initiates lipid peroxidation of cellular membranes, particularly affecting polyunsaturated fatty acids in cell walls and organelles. This irreversible oxidative damage is biochemically marked by elevated malondialdehyde (MDA) levels—a terminal byproduct of membrane lipid degradation that directly correlates with cellular necrosis [20]. Consequently, MDA serves as a key biomarker quantifying the collapse of membrane integrity during pathogenic assault, linking initial ROS signaling to ultimate tissue maceration in the plant-pathogen battleground.

Thus, tracking the fluctuations of SOD, POD, and MDA not only illustrates the physiological reactions of kiwifruit to pathogen invasion but also serves as vital markers for identifying disease-resistant varieties. Nonetheless, existing studies mainly concentrate on identifying pathogens and employing chemical treatments [8,9,21], resulting in considerable gaps in our understanding of the physiological responses of the fruit to disease resistance. Therefore, this research focused on the highly virulent strain *Diaporthe eres* P3-1W, conducting a systematic analysis for the first time of the dynamic variations in SOD, POD, and MDA in Hongyang kiwifruit throughout the infection process. And the expression patterns of SOD/POD-encoding genes and the correlationship among these genes were also analyzed in this study. The objective is to offer theoretical insights for the selection of disease-resistant germplasm and the advancement of targeted preservation methods.

## 2. Materials and Methods

### 2.1. Plant Material and Pathogen

Fruits of the ‘Hongyang’ kiwifruit variety (*A. chinensis* cv. *Hongyang*) of comparable dimensions were acquired from a nearby market in Liupanshui, Guizhou Province. The pathogen *D. eres* P3-1W was used in this study, which was isolated and identified from rotting ‘Hongyang’ kiwifruit in our prior investigation [22].

### 2.2. Pathogen Inoculation and Sampling

The *D. eres* P3-1W strain was grown in darkness at 25 °C on potato dextrose agar (PDA) for a duration of three days. A mycelial plug measuring 5 mm in diameter was then utilized to inoculate kiwifruit, which had been sterilized on the surface and wounded using a five-hole technique prior to the inoculation. For the control group, a sterile PDA plug of the same size was used. The inoculated kiwifruits were placed in sealed containers and kept in the dark. Tissue samples were taken from the boundary between diseased and healthy regions at intervals of 0, 1, 2, 3, 4, and 5 days after inoculation (dpi). These samples were promptly frozen in liquid nitrogen and stored at −80 °C for physiological analysis. Additionally, the progression of lesion size was monitored and documented through photographs. Three separate biological replicates were conducted for each time point.

### 2.3. Enzyme Activity Assays

#### 2.3.1. SOD Activity Assay

Crude enzyme extract was obtained by homogenizing tissue samples (0.5 g fresh weight, FW) in ice-cold 0.05 mol/L phosphate buffer (pH 7.8) followed by centrifugation (10,000 rpm, 15 min, 4 °C). SOD activity in the supernatant was determined spectrophotometrically based on its ability to inhibit the photochemical reduction of nitroblue tetrazolium (NBT). A control reaction replaced the enzyme extract with buffer. Reaction tubes were incubated for 30 min under controlled light conditions to initiate riboflavin-mediated superoxide generation and NBT reduction. Absorbance was recorded at 560 nm. One unit of SOD activity (U) was defined as the quantity of enzyme needed to reduce NBT reduction by 50% under the given assay conditions, with the enzyme activity reported per gram of fresh weight.

#### 2.3.2. POD Activity Assay

Crude enzyme extract was prepared by homogenizing tissue samples (0.5 g fresh weight) with polyvinylpolypyrrolidone (PVPP) in ice-cold 0.1 mol/L phosphate buffer (pH 6.8), followed by centrifugation (10,000 rpm, 15 min, 4 °C). POD activity in the supernatant was determined spectrophotometrically by measuring the oxidation of guaiacol. A control reaction replaced the enzyme extract with buffer. The increase in absorbance at 470 nm, reflecting the formation of oxidized guaiacol (tetraguaiacol), was monitored immediately after mixing for 1–3 min at room temperature. Enzyme activity (U) was defined as the amount causing a ΔA470 of 0.01 per minute under the assay conditions and expressed per gram of fresh weight or milligram of protein, as appropriate. This study included three independent biological replicates for the activities of SOD and POD.

### 2.4. Measurement of Malondialdehyde (MDA) Content

MDA concentration, an indicator of lipid peroxidation, was quantified using the thiobarbituric acid (TBA) assay. Tissue samples (0.2 g fresh weight) were homogenized in trichloroacetic acid (TCA) solution. The homogenate was centrifuged, and the supernatant (sample extract) was reacted with TBA solution. A control reaction replaced the sample extract with distilled water. The reaction mixtures were heated to form the MDA-TBA adduct, cooled, and absorbance measured at 532 nm, 600 nm (to correct for non-specific turbidity), and 450 nm (to correct for sucrose interference). MDA concentration was calculated using a standard formula incorporating absorbance corrections at these three wavelengths. The final MDA content is expressed as micromoles per gram of fresh weight (μmol/g FW), adjusted based on the initial sample weight and reaction volumes.

### 2.5. Transcriptome Sequencing and Analysis

A total of 15 fruit tissue samples were collected at 0, 12, 24, 48, and 72 h post-infection (hpi). Total RNA was extracted from these samples and used for library construction, followed by sequencing on the BGIseq-500 platform. Clean reads were mapped to the kiwifruit reference genome (release-46_Red5_PS1_1.69.0) using Bowtie2 [23]. Gene annotation was performed by aligning sequences to public databases (Nr, Gene Ontology [GO], and Kyoto Encyclopedia of Genes and Genomes [KEGG]). SOD- and POD-encoding genes were identified based on annotations from these databases, selecting those exhibiting >2-fold expression change between any two time points. Transcript abundance was quantified as fragments per kilobase of exon per million mapped fragments (FPKM). Time-series expression patterns were clustered using the Mfuzz package in R (version 4.5.1). Pairwise Pearson correlation coefficients were calculated both between genes and across all samples. The genes encoding SOD and POD were identified based on the annotation of three public databases and the fold change in expression level between two time points was more than 2. The raw sequencing data from this study have been deposited in the BIG Data Center GSA database (Accession No. PRJCA039689).

### 2.6. Bioinformatic Analysis

Signal peptide prediction and subcellular localization were conducted utilizing the online tools SignalP 5.0 (https://services.healthtech.dtu.dk/services/SignalP-5.0/, accessed on 16 August 2025) and CELLO v2.5 (http://cello.life.nctu.edu.tw/, accessed on 16 August 2025). The analyses of secondary and tertiary structures of SOD/POD proteins were performed using the online tools SOPMA (https://npsa-prabi.ibcp.fr/cgi-bin/npsa_automat.pl?page=/NPSA/npsa_sopma_f.html, accessed on 16 August 2025) and SWISS-MODEL (https://swissmodel.expasy.org/, accessed on 16 August 2025).

### 2.7. Statistical Analysis

The software SPSS 20.0 was utilized to analyze the data and assess the significance of differences, with a threshold set at *p* < 0.05. Results were presented as mean values accompanied by standard error.

## 3. Results

### 3.1. Disease Progression and Incubation Period Dynamics

**Figure 1:**
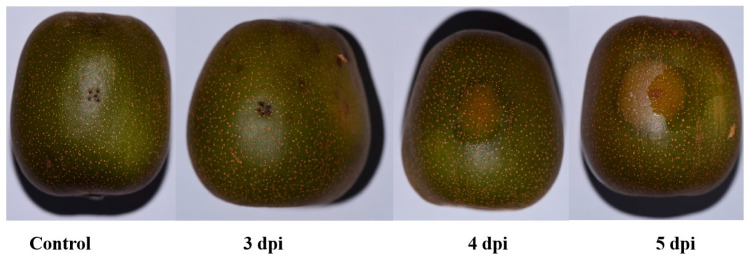
Symptom development in Hongyang kiwifruit inoculated with *Diaporthe eres* at different time points.

In this study, we first observed the disease progression at the different incubation periods (0–5 days). We found that the symptoms on Hongyang kiwifruit infected with *D. eres* were first noted three days after inoculation (dpi) (Figure 1). The disease’s advancement was marked by increasingly severe soft rot symptoms by four dpi. At this point, the edges of the lesions showed typical water-soaking and tissue leakage (Figure 1). These observations suggest that the incubation period for *D. eres* in Hongyang kiwifruit mainly takes place during the initial three days following inoculation. Although there are no clear external signs during this time, the pathogen successfully colonizes and begins to spread within the fruit’s flesh. The appearance of exudative lesions at four dpi indicates that the affected tissue has entered a stage of irreversible cellular breakdown. This deterioration involves damage to the membrane systems, leading to the release of intracellular materials and the development of water-soaked symptoms.

### 3.2. Bimodal SOD Activation Patterns in Diaporthe-Infected Hongyang Kiwifruit

**Figure 2:**
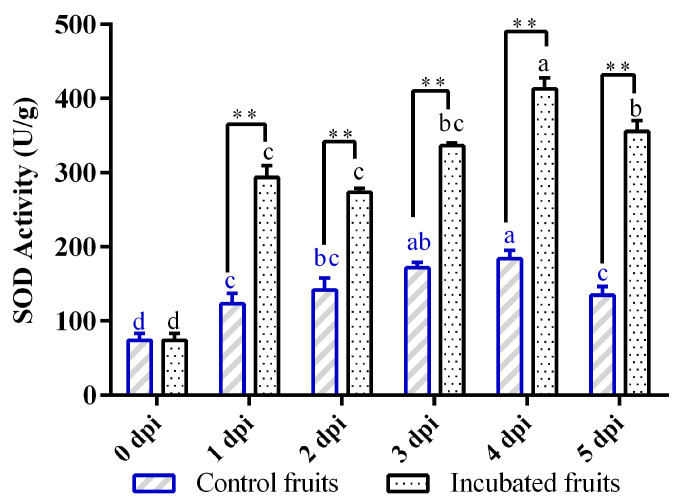
Changes in superoxide dismutase (SOD) activity in Hongyang kiwifruit inoculated with *Diaporthe eres* at different time points. Note: Different letters in each line with the same color indicate significant difference at the different timepoints (*p* < 0.05); two asterisks (**) indicate the significant difference of incubated fruits and control fruits (*p* < 0.01).

Infection by *Diaporthe* triggered a notable bimodal response in the activity of SOD (Figure 2). In the early stages of infection (0–2 dpi), there was a dramatic rise in enzyme activity that peaked at 1 dpi (*p* < 0.01 compared to controls during the same timeframe). Although there was a slight, non-significant drop at 2 dpi (Figure 2), this initial surge indicated a swift mobilization of SOD in Hongyang kiwifruit to eliminate superoxide anion radicals (O_2_^−^) and mitigate oxidative stress caused by the pathogen. A more significant secondary peak was observed at 4 dpi, which was 2.25 times higher than the controls (183.82 U/g; *p* < 0.01) (Figure 2). This increase suggested the activation of a more profound defense mechanism to address the rapid growth of the pathogen. Control fruits displayed an escalating trend and reached a peak on day 4 post-injury before experiencing a decline (Figure 2). Overall, this sequential activation illustrated a layered defense approach in Hongyang kiwifruit: an immediate response to ROS followed by a heightened antioxidant response to manage the advancing infection.

### 3.3. Temporal Changes in POD Activity During Diaporthe-Infected Hongyang Kiwifruit

**Figure 3:**
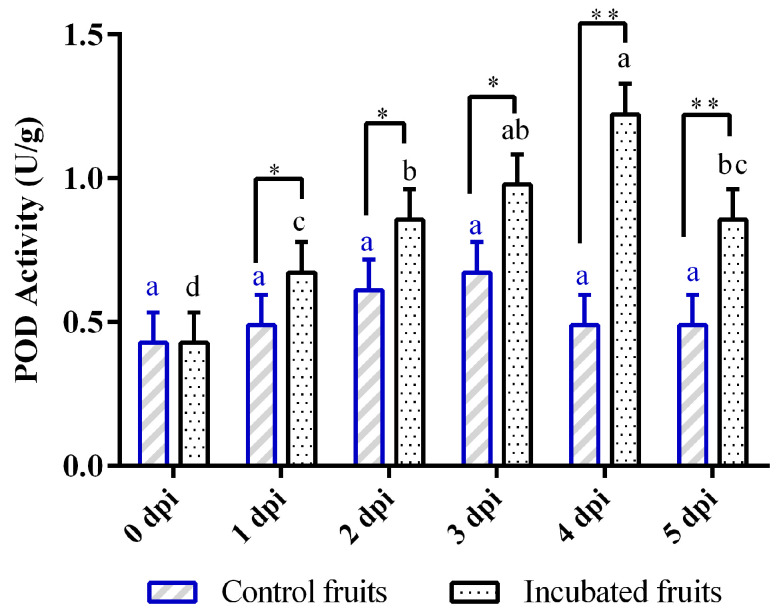
Changes in peroxidase (POD) activity in Hongyang kiwifruit inoculated with *Diaporthe eres* at different time points. Note: Different letters in each line with the same color indicate significant difference at the different timepoints (*p* < 0.05); one asterisk (*) indicates the significant difference of incubated fruits and control fruits (*p* < 0.05); two asterisks (**) indicate the significant difference of incubated fruits and control fruits (*p* < 0.01).

Peroxidase (POD) plays a crucial role in the defense mechanisms of plants and often shows notable changes in activity when faced with pathogen attacks. In this study, the POD activity exhibited a significant increase when Hongyang kiwifruit was infected with *Diaporthe eres* at 1 dpi (Figure 3). It reached its highest level on the fourth dpi, which was significantly higher than the control (*p* < 0.01). However, its activity apparently decreased by the fifth dpi (Figure 3). In contrast, the POD levels in the control kiwifruits remained fairly constant throughout the study, with no significant variations observed (*p* > 0.05) at different time intervals (Figure 3).

### 3.4. Dynamics of Malondialdehyde (MDA) Accumulation

**Figure 4:**
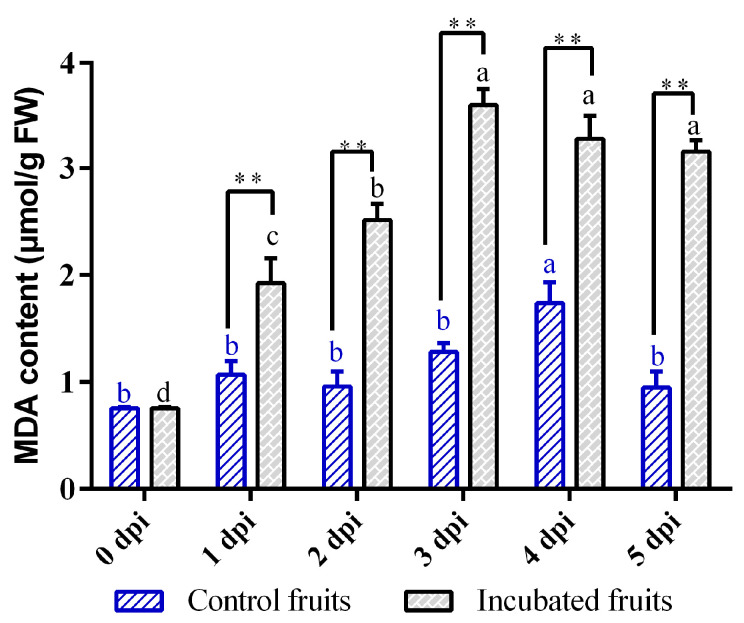
Changes in malondialdehyde (MDA) content in Hongyang kiwifruit inoculated with *Diaporthe eres* at different time points. Note: Different letters in each line with the same color indicate significant difference at the different timepoints (*p* < 0.05); two asterisks (**) indicate the significant difference of incubated fruits and control fruits (*p* < 0.01).

MDA serves as a crucial indicator of oxidative stress in plants facing challenging conditions, showing unique temporal patterns in kiwifruit infected by *Diaporthe* (Figure 4). The infection initiated a rapid accumulation phase of MDA levels within the first three dpi, marking a 374% increase that significantly surpassed the levels observed in the control group at 3 dpi (*p* < 0.01). Although MDA levels slightly decreased after 3 dpi, they remained elevated with minimal daily fluctuations (*p* > 0.05). In contrast, the control kiwifruits exhibited consistent MDA levels throughout the study, aside from a brief spike (1.28 μmol/g) on the fourth day of wounding, followed by a decline (Figure 4). Overall, these findings indicate that *Diaporthe eres* infection leads to a significant and prolonged increase in cellular MDA in kiwifruit, with peak oxidative membrane damage occurring by 3 dpi.

### 3.5. Transcriptome Analysis of the SOD and POD Genes

In this study, we identified a total of 8 SOD genes and 19 POD genes (detailed information in Table S1) in the latent period of pathogen infection (0–3 dpi). Analysis revealed that the majority of the SOD genes belong to the Cu/Zn-SOD family (e.g., *CEY00_Acc22834*), with only two (*CEY00_Acc02790* and *CEY00_Acc03641*) classified as Mn-SODs (Table S1). Most SOD genes exhibited peak expression levels at 24 h post-inoculation (hpi). However, the expression of three SOD genes continued to increase beyond 24 hpi (Figure S1). Notably, *CEY00_Acc02790* displayed consistently high expression levels throughout all infection stages, reaching its maximum at 72 hpi (Figure S1). Cluster analysis classified the SOD genes into two distinct groups (Cluster 1 and Cluster 2) based on their expression patterns (Figure S2).

In contrast, POD genes exhibited greater diversity in their expression dynamics. Following pathogenic fungal inoculation, expression levels of five POD genes decreased, while others increased; the remaining POD genes showed an initial increase followed by a decrease (Figure S3). Consequently, these POD genes were clustered into three distinct groups based on their expression profiles (Figure S2). Among these, *CEY00_Acc11608* exhibited the highest expression levels at each infection time point.

**Figure 5:**
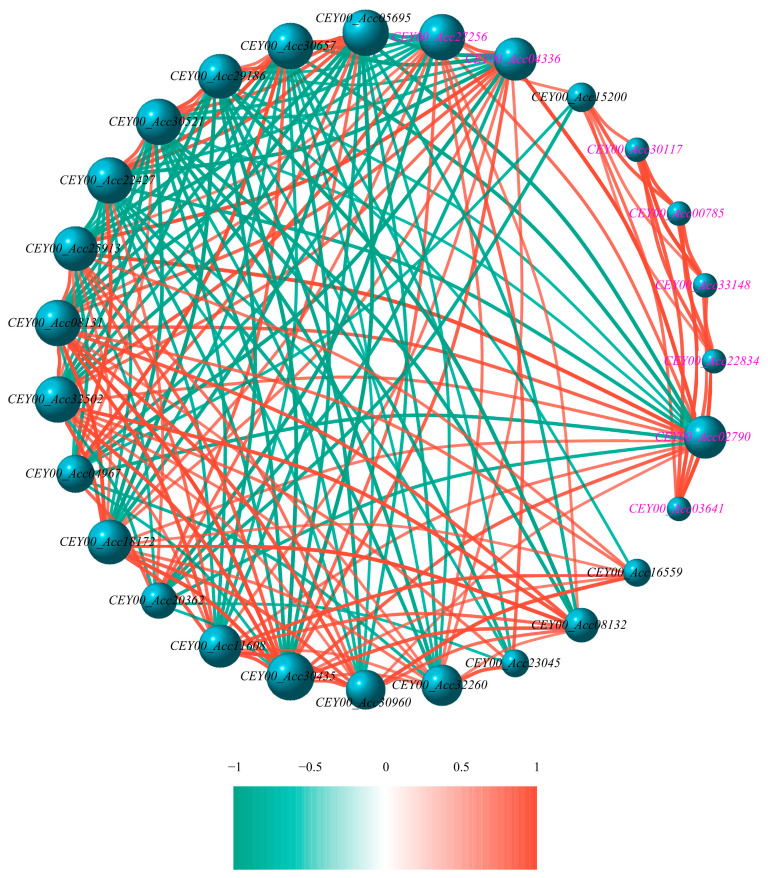
The correlation analysis of SOD and POD genes. Note: The genes in pink and black font indicate SOD and POD genes, respectively.

Network analysis of all SOD and POD genes identified co-expression hubs. Within these hubs, regulatory interactions between SOD genes were limited. However, three SOD genes (*CEY00_Acc02790*, *CEY00_Acc04336*, and *CEY00_Acc27256*) were found to regulate POD gene expression, exhibiting both positive and negative regulatory effects (Figure 5). Conversely, the POD genes themselves displayed regulatory relationships, with some predominantly exerting negative regulation and others positive regulation on their expression network (Figure 5).

### 3.6. Bioinformatic Analysis of CEY00_Acc02790 Regulatory Network

**Figure 6:**
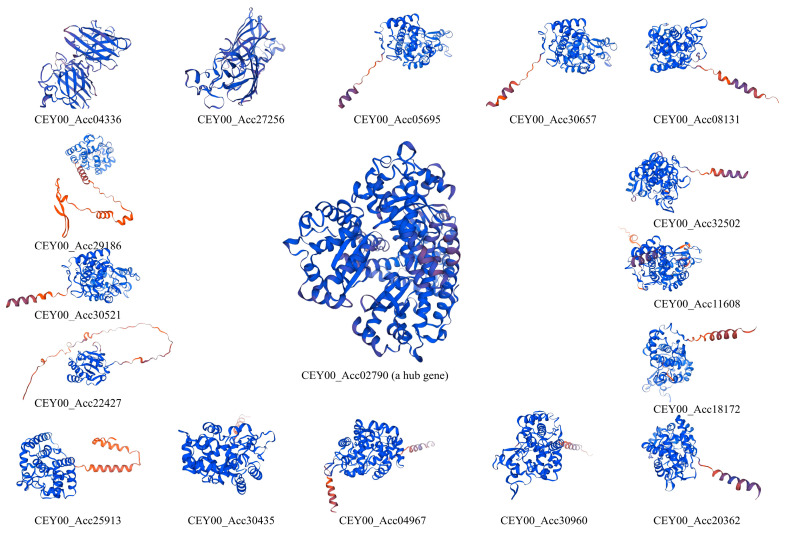
The protein tertiary structure prediction of *CEY00_Acc02790* and its co-expressed genes.

To further understand the functions of these genes, we analyzed the network of *CEY00_Acc02790* as a hub gene using bioinformatics tools. In this regulatory network, *CEY00_Acc02790* was found to have seven negative and nine positive co-regulatory genes (Figure 5). Among these, only *CEY00_Acc04336* and *CEY00_Acc27256* were classified as SOD genes. Subcellular localization predictions using CELLO v2.5 software indicated that 11 proteins were primarily located outside the cytoplasm, two were found in the chloroplast, and three were localized within the cytoplasm, with *CEY00_Acc02790* specifically localized in the mitochondria (Table S2). Signal peptide prediction analysis conducted with SignalP 5.0 revealed that all 11 extracellular proteins contained signal peptides, while the remaining proteins did not (Table S2). The secondary structure prediction results indicate that all gene-encoded proteins lacked β-sheets and were predominantly composed of α-helices, random coils, extended chain structures, and β-turns. The α-helix contents for *CEY00_Acc04336* and *CEY00_Acc27256* were 9.21% and 5.92%, respectively, while their random coil contents were 50% and 53.29%. The proportions of extended chain structures and β-turns were consistently measured at 32.89% and 7.89%, respectively. For *CEY00_Acc02790*, the values of these four structural components for the SOD genes were 52.84%, 12.23%, 6.55%, and 28.38%, respectively. The α-helix content among the 14 POD genes ranged from 20.99% to 43.23%; the random coil content ranged from 37.94% to 48.56%; the extended strand structure ranged from 11.88% to 21.81%; and the β-turn content ranged from 4.02% to 8.91%. Figure 6 clearly illustrates the spatial structures of α-helices, β-turns, extended strands, and random coils for the three SOD proteins and the 14 POD proteins. The varying proportions of α-helices, β-turns, extended strands, and random coils among the proteins contribute to differences in their spatial structures and functional differentiation. Additionally, the Global Model Quality Estimation (GMOE) values for all 17 proteins exceed 0.80, except for *CEY00_Acc29186*, which has a value of 0.74, indicating a high reliability of these structural predictions (Table S2).

## 4. Discussion

*Diaporthe eres* serves as a primary causal agent of postharvest soft rot in Hongyang kiwifruit [22]. This study delineates the dynamic responses of key oxidative stress markers—SOD, POD, and MDA—and their integrated defense mechanisms during pathogenesis. Our results confirm an approximately 3-day latent period (0–3 dpi), consistent with a recent study where pathogens remain undetectable before triggering rapid tissue maceration [24]. This timeframe aligns with observations of GFP-labeled *D. actinidiae* hyphae proliferating within fruit tissue as early as 1 dpi [25], collectively indicating that *D. eres* establishes colonization and initiates proliferation during this subclinical phase. The subsequent emergence of water-soaked lesions and tissue exudation at 4 dpi signifies irreversible cellular breakdown, temporally coinciding with marked alterations in oxidative stress markers. This transition aligns with the demonstrated requirement for ROS in activating programmed cell death (PCD) during the hypersensitive response (HR) to pathogen invasion [26].

ROS production serves as a critical defense component in both pattern-triggered immunity (PTI) and effector-triggered immunity (ETI) [27]. During *D. eres* infection (0–5 dpi), Hongyang kiwifruit mounted a bimodal response in SOD activity. An initial surge in SOD activity was observed at 1 dpi, suggesting a rapid mobilization of antioxidant defense to effectively scavenge O_2_^−^ radicals generated by pathogen invasion. Our transcriptome findings revealed that the majority of SOD genes exhibited their highest expression levels at 1 dpi, corresponding temporally with the SOD activity. A secondary peak in SOD activity at 4 dpi further indicated a sustained and enhanced defense response to manage the advancing infection. SOD is often reported as a key antioxidant enzyme in the initial defense response in many plants [17,27]. However, the bimodal pattern observed in Hongyang kiwifruit might be more pronounced than in some other plant-pathogen interactions, suggesting a more complex or prolonged defense response in this specific host–pathogen system.

Peroxidases are one of the first antioxidant enzymes, which play a role in lignification, phenylpropanoid metabolism, and the production of antimicrobial compounds, which are key components of plant defense mechanisms [28,29]. In this study, POD activity increased synchronously (55.8% at 1 dpi) with SOD activity and showed a sustained increase peaking at 4 dpi. However, the transcriptome revealed a more complex regulation of POD genes compared to SOD. This transcriptional heterogeneity suggests nuanced regulatory mechanisms governing POD isoenzymes, potentially fine-tuning H_2_O_2_ metabolism in different cellular compartments or in response to specific signals during infection. Our results revealed that 11 POD possessed signal peptides and localized extracellularly, which were characteristically high α-helix content (20.99–43.23%) and extended strand proportions (11.88–21.81%). A recent study reported that H_2_O_2_ oxidizes transcription factor bHLH25 to reinforce cell walls through lignin cross-linking against pathogen-derived cell wall-degrading enzymes (CWDEs) [30]. In addition, our previous study found that several CWDEs (PG, PME, Cx) exhibited the peak activities during early infection (1–2 dpi) [22]. Therefore, these POD isoenzymes might confer rigid substrate-binding pockets optimal for polymerizing bulky phenolic compounds during defensive lignification. In contrast, three of POD dominating by random coils (37.94–48.56%) lacked signal peptides and localized intracellularly, which might function as H_2_O_2_ scavengers to protect cytoplasmic components from oxidative damage [28]. The functional diversity of POD in plant defense against fungal pathogens underscores the importance of multiple enzymatic pathways in host defense [29,31]. The transcriptome data of these POD genes exhibited the diversity of the expression patterns. However, the specific role of POD in Hongyang kiwifruit against *Diaporthe* remains to be further investigated.

**Figure 7:**
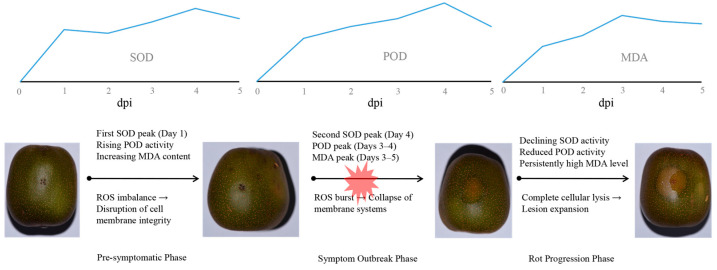
Modeling the phased antioxidant defense dynamics in *Diaporthe eres*-kiwifruit pathosystem.

Previous studies demonstrate that ETI generates a stronger and more sustained ROS burst than PTI [32,33], with H_2_O_2_ production being essential for HR-mediated cell death that restricts pathogen spread [34]. Our findings reveal that the biphasic SOD activation reflects a staged defense strategy (Figure 7). As infection advanced, the antioxidant capacity progressively weakened. Despite SOD reaching peak activity at 4 dpi, exponential pathogen proliferation likely overwhelmed ROS-scavenging capabilities. Concurrently, POD activity declined significantly by 5 dpi, signaling systemic antioxidant collapse. The 4.75-fold MDA surge at 3 dpi denotes severe membrane compromise preceding cellular failure. Thus, while the SOD-driven initial response temporarily impedes pathogen spread, sustained biotic pressure ultimately overwhelms defense mechanisms, culminating in membrane system failure and fruit decay (Figure 7). Moreover, our co-expression network analysis provided molecular evidence supporting the functional coordination between SOD and POD. Therefore, both of them might establish a SOD-POD defense cascade, synergistically decomposing H_2_O_2_ to mitigate oxidative damage. This coordinated enzymatic response indicates that pathogen-associated molecular pattern (PAMP) recognition might trigger substantial ROS production during initial defense activation in kiwifruit just like in other plants [35]. Collectively, Hongyang kiwifruit might deploy a phased, multi-layered defense strategy centered on SOD-POD coordination, with MDA serving as a biomarker of progressive membrane integrity loss. Further research should elucidate the biochemical signaling and physiological regulation governing these defense responses to pathogen invasion.

## 5. Conclusions

This study delineates the phased antioxidant defense strategy in Hongyang kiwifruit against *Diaporthe eres* infection, identifying pivotal targets for postharvest protection. The 3-day symptom-free colonization window (0–3 dpi) provides a critical timeframe for preemptive intervention, while the mitochondrial SOD hub gene *CEY00_Acc02790* orchestrates early defense through the coordinated regulation of POD genes. Bioinformatic analysis revealed the functional specialization of POD isoforms: α-helix-rich extracellular variants may drive cell wall reinforcement via lignification, while random coil-dominant intracellular isoforms may mitigate cytoplasmic ROS damage, suggesting dual barriers against pathogenesis. Crucially, MDA accumulation by 3 dpi serves as a quantifiable biomarker for early prediction of irreversible membrane compromise. These findings provide fundamental insights into the *Actinidia*–*Diaporthe* pathosystem, establishing that the failure of the phased SOD-POD defense axis precedes symptom outbreak and guiding the optimization of storage protocols, extending shelf-life by disrupting pathogenesis before visible decay.

## Data Availability

The raw sequencing data from this study have been deposited in the BIG Data Center GSA database (Accession No. PRJCA039689).

## Supplementary Materials

The following supporting information can be downloaded at https://www.mdpi.com/article/10.3390/biology14091169/s1: Figure S1: The expression analysis of SOD genes across each infection stage; Figure S2: Hierarchical clustering analysis of all SOD and POD genes; Figure S3: The expression analysis of POD genes across each infection stage; Table S1: Information of SOD and POD genes identified in Hongyang kiwifruit during infection; Table S2: The properties and structural information of the co-expression network of *CEY00_Acc02790* as a hub gene.
