# Supplementary figures and images for: Antioxidant Defense Strategies Against Diaporthe eres Infection in Hongyang Kiwifruit
Source: Biology (Basel). 2025 Sep 2;14(9):1169. doi: 10.3390/biology14091169 (PMC12467673; doi:10.3390/biology14091169)

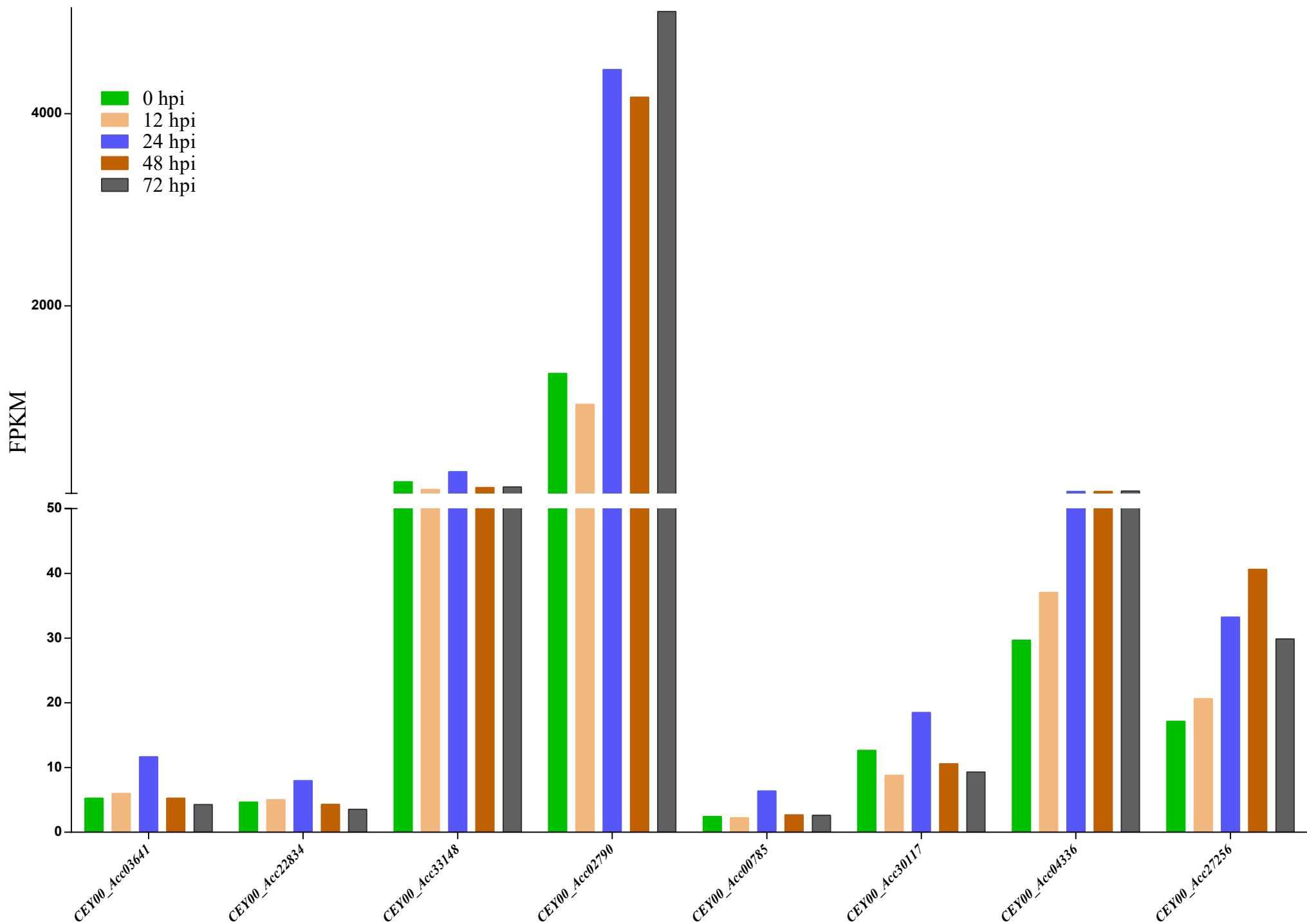

Supplement: Supplementary file 1 [file biology-14-01169-s001.zip › Figure S1.pdf]

# Cluster 1

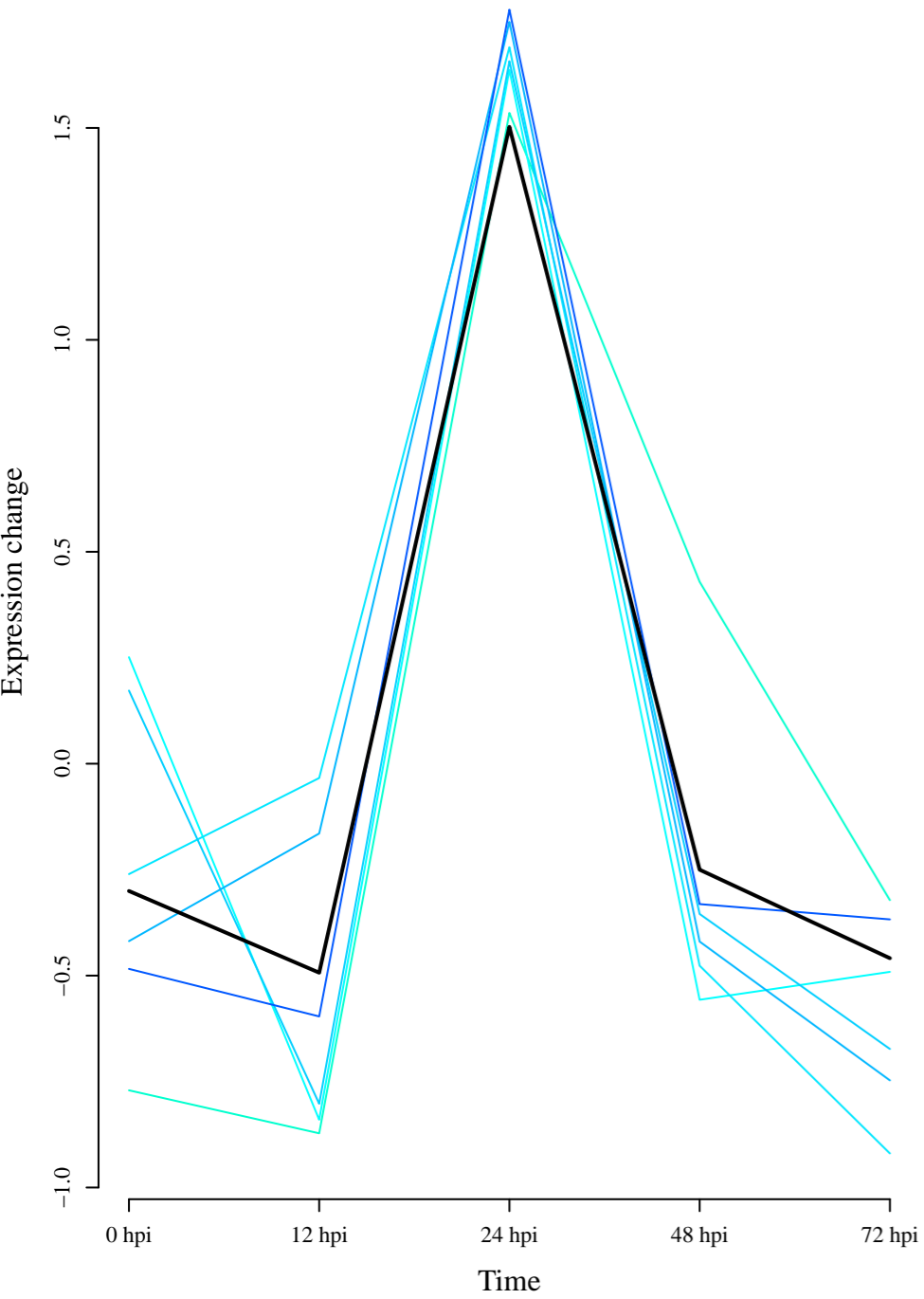

# Cluster 2

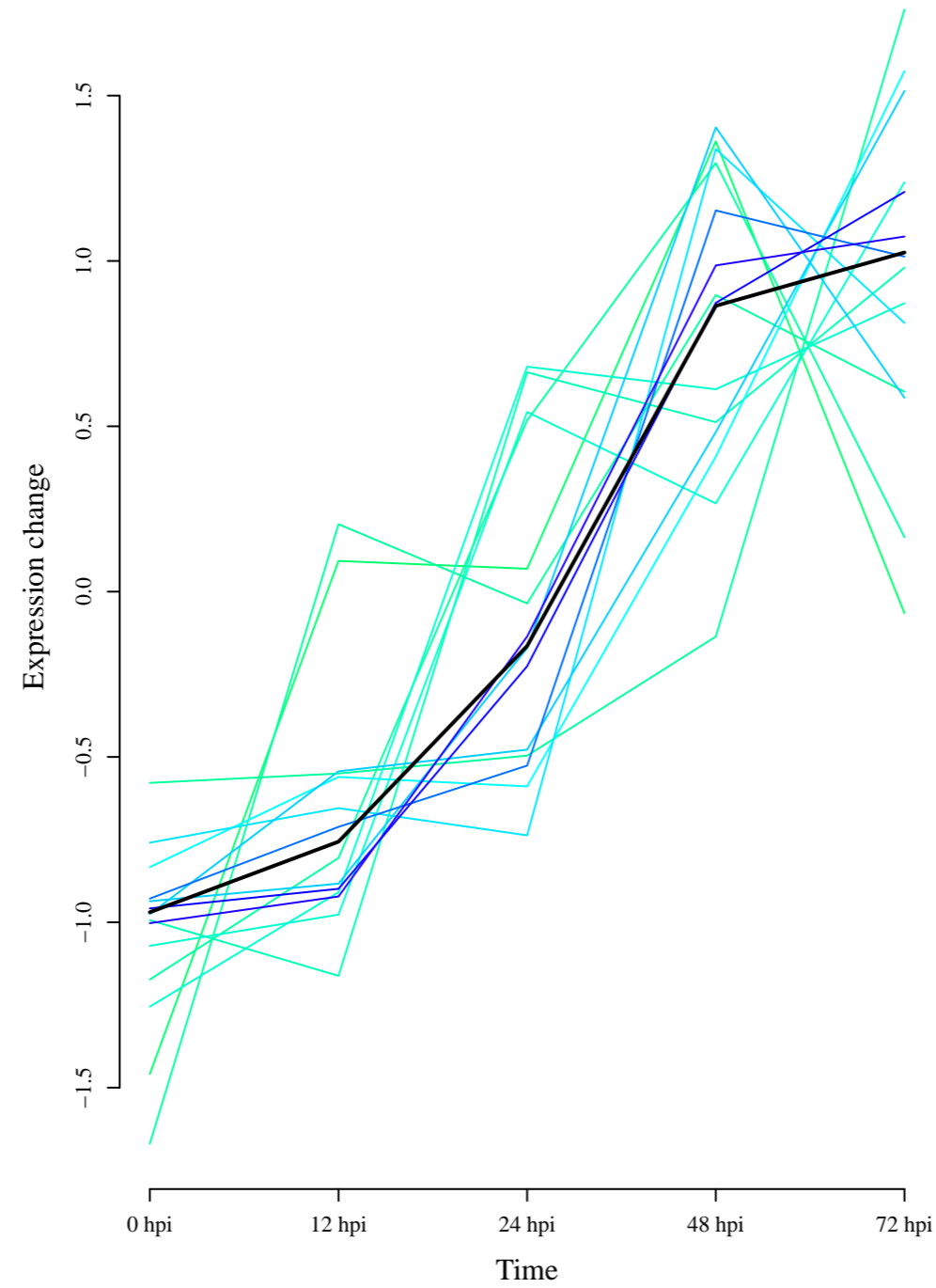

# Cluster 3

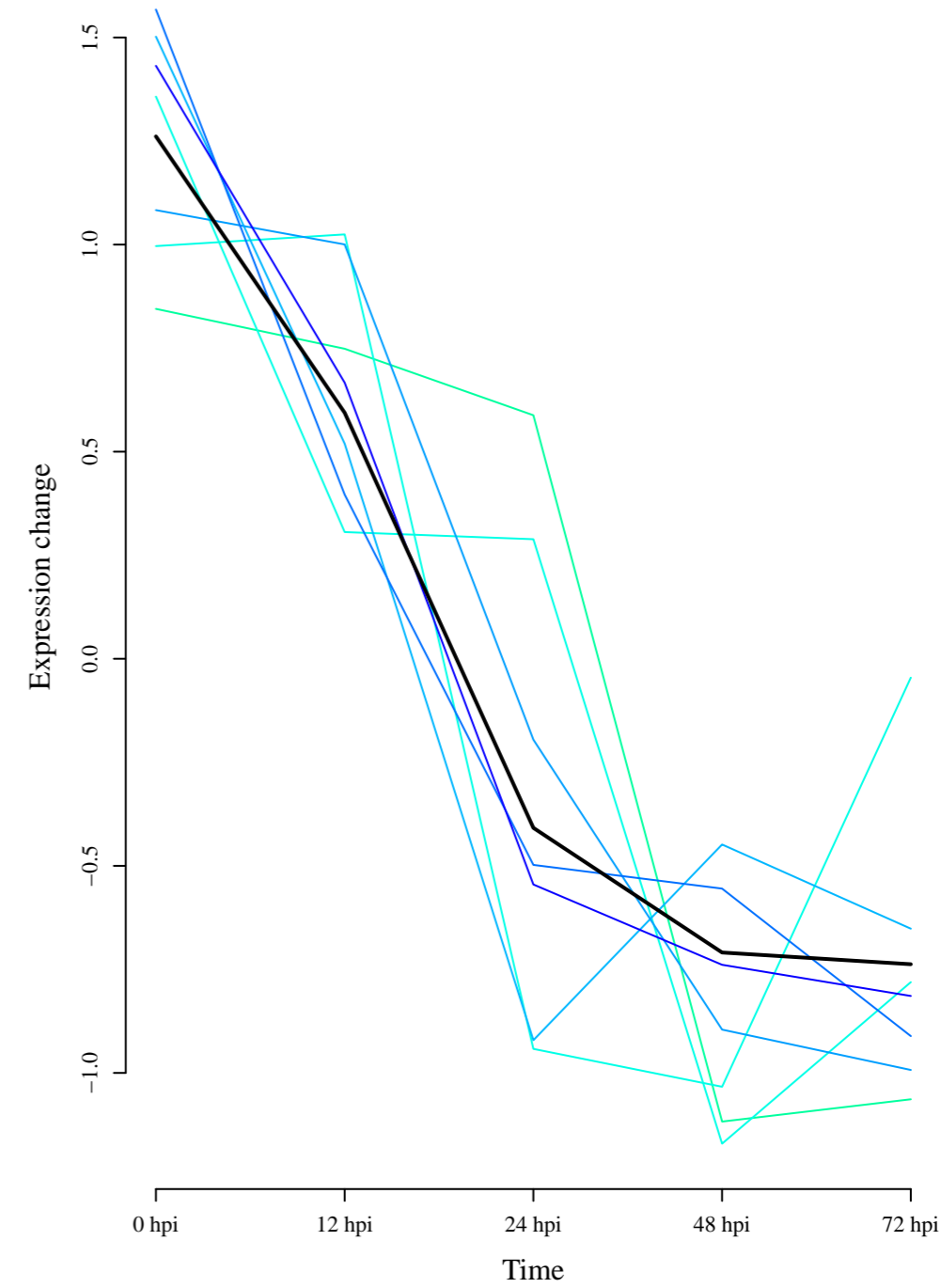

Membership

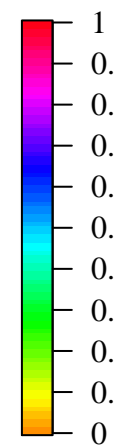

Supplement: Supplementary file 1 [file biology-14-01169-s001.zip › Figure S2.pdf]

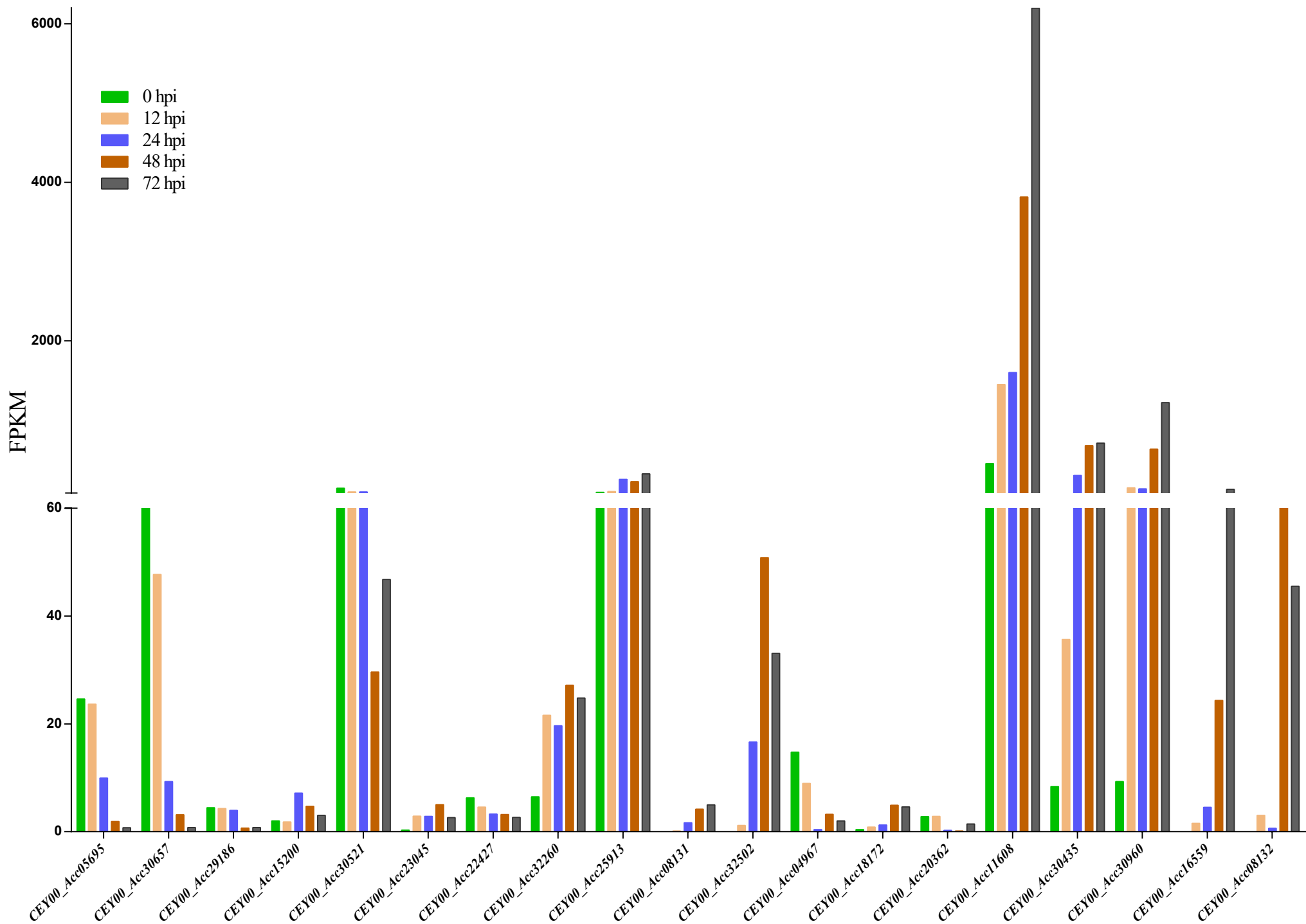

Supplement: Supplementary file 1 [file biology-14-01169-s001.zip › Figure S3.pdf]
